# Better soils for healthier lives? An econometric assessment of the link between soil nutrients and malnutrition in Sub-Saharan Africa

**DOI:** 10.1371/journal.pone.0210642

**Published:** 2019-01-17

**Authors:** Ezra D. Berkhout, Mandy Malan, Tom Kram

**Affiliations:** 1 PBL Netherlands Environmental Assessment Agency, The Hague, The Netherlands; 2 International Institute of Tropical Agriculture (IITA), Kampala, Uganda; Ohio State University South Centers, UNITED STATES

## Abstract

Malnutrition, the suboptimal consumption of essential nutrients like zinc, severely affects human health. This burden of malnutrition falls disproportionally heavy on developing countries, directly increasing child mortality and childhood stunting, or reducing people’s ability mending diseases. One option to combat malnutrition is to blend missing nutrients in crop fertilizers, thereby increasing crop yields and possibly the nutrient density in harvested crop products, thus enriching crop products destined for human consumption. But, the effectiveness of so-called agronomic fortification remains ill-understood, primarily due to a paucity of field trials. We hypothesize that, if at all this is an effective strategy, there should exist a causal link between malnutrition and natural variation in the quality of soils to begin with. Until now, data limitations prevented the establishment of such a link, but new soil micronutrient maps for Sub-Saharan Africa allow for a detailed assessment. In doing so, we find statistically significant relations between soil nutrients and child mortality, stunting, wasting and underweight. For instance, a simultaneous increase in soil densities of copper, manganese and zinc by one standard deviation reduces child mortality by 4–6 per mille points, but only when malaria pressure is modest. The effects of soil nutrients on health dissipate when malaria pressure increases. Yet, the effects are fairly small in magnitude suggesting that except for a few regions, agronomic fortification is a relatively cost ineffective means to combat malnutrition.

## Introduction

Malnutrition–also dubbed hidden hunger–refers to a suboptimal intake of essential nutrients (proteins, minerals, metals and vitamins), even if and where calorific intake is at least sufficient. Globally, 2–3 billion people are estimated to be deficient in micronutrients, mostly iodine, iron (60% of the global population) and zinc (30% of the global population) [1, 2]. Zinc deficiency prolongs (a.o.) episodes of diarrhoea leading to dehydration and is a leading cause of child mortality [3]. Deficiencies are also associated with childhood stunting (height of child too low for age) and wasting (weight of child too low for height), impairing child cognitive and physical development [4]. This burden of malnutrition is particularly severe in developing societies, including most countries in Sub-Saharan Africa (SSA) [3, 5]. Moreover, ill-health carries a considerable economic penalty. It is thought that zinc and iron deficiencies reduce GDP of developing countries by 2–5% [5].

For these reasons, alleviating malnutrition is widely considered a top priority in development and development assistance. The Copenhagen Consensus Center [6], for instance, consistently lists alleviating malnutrition as one of the most cost-efficient development interventions (e.g. [7, 8]). By and large, five different interventions exist to address malnutrition. Three of these are post-harvest interventions: 1) the fortification of commonly consumed, processed foods, 2) supplementation in the form of powders or capsules and 3) the promotion of dietary diversity. The others, 4) genetic fortification and 5) agronomic fortification, fall within the realm of agricultural production and/or agricultural development assistance. The potential of agronomic fortification in SSA is the focus of this study.

Our study is motivated by the observation that relatively little information exists on the cost-effectiveness of these agriculture-based interventions in order to guide policy-makers in the choice for one, or combinations of these. Most insights into cost-effectiveness as put forward by the Copenhagen Consensus are based on food fortification or supplementation [8]. Ex-ante assessments of genetic fortification–breeding new crop varieties with higher nutrient content–do suggest it can be highly cost-effective [9], but very few ex-post evaluations exist to date. Agronomic fortification entails the supplementation of micronutrients through inorganic fertilizer. The rationale is that micronutrient deficiencies in food may stem from inherently low densities in the harvested products, caused by missing nutrients in soils. The latter also provides a partial explanation for low agricultural productivity in SSA itself [10]. In fact, genetic fortification may be an inefficient strategy when soil nutrient densities are low to begin with [11]. Moreover, the promotion of organic fertilizers, sourced locally in nutrient scarce regions, is likely to be insufficient for addressing soil nutrient deficiencies. The latter necessitates the supply of nutrients from external sources.

Agronomic fortification could thus be one stone to kill two birds, raising both agricultural productivity and production, as well as reducing incidences of malnutrition. Various scientists and organizations, not least the International Zinc Association (IZA), point to these perceived dual benefits [12–15]. But, little information is available to understand its actual impact and cost-effectiveness. This stems from a paucity of field trials, particularly in Sub-Saharan Africa. A recent systematic review for SSA [16], identifying only forty studies, indeed reveals a positive impact of micronutrient-enriched fertilizers on agricultural productivity. One study [12] does assess the ex-ante impact of agronomic fortification with zinc on human nutritional status, and suggests it can be cost-effective. But, to our knowledge, no studies have assessed impact of agronomic fortification on human nutritional status in Sub-Saharan Africa ex-post. Altogether, we know of only one detailed study documenting the impact of agronomic fortification on human health, being one on selenium-enriched fertilizers in Finland [17].

Whether or not agronomic fortification is a cost-effective means to alleviate the burden of malnutrition thus remains ill-understood. To circumvent this information scarcity this study follows a different approach. We hypothesize that if, at all, enriching soils with supplemental (micro-)nutrients has a beneficial effect on human health, such a relation should also become apparent when assessing the impact of natural variation in soil nutrient densities on human health. In other words, do differences in health outcomes become apparent and statistically significant when comparing two regions that differ with respect to average densities of soil nutrients only (or when differences are otherwise controlled for)? If so, we hypothesize that the magnitude of this effect provides a fairly robust proxy for the potential impact of agronomic fortification.

To our knowledge, such a relation between soil nutrients and human nutritional status has not been assessed before, mostly due to limited availability of soil data. This study, however, builds on a novel dataset that maps predicted nutrient densities for an array of macro- and micronutrients across Sub-Saharan Africa at a high spatial resolution [18]. We subsequently estimate several econometric model specifications in order to discern the causal effects of variation in such densities on spatial differences in child malnutrition (mortality, stunting, wasting and underweight). We find a number of statistically significant effects, for some nutrients on some of these health indicators. Mostly, the signs of these effects are plausible and as expected: increases in micronutrients manganese, zinc and copper translate into reductions in child mortality and stunting. Children living in areas with soils rich in calcium and magnesium are typically taller, thus reducing incidences of stunting but, all other things being equal, increasing wasting.

In the final step of this paper we use the estimated magnitude of the effect of additional soil micronutrients on child mortality to calculate the costs per child life saved and compare this figure with cost-benefits of other interventions. Even though the effects are statistically significant the actual magnitude of the effect is fairly small. This translates into costs per child life saved that are often greater than those of other alternative strategies available. The exceptions are regions where both population densities are high, soil nutrient densities and malaria pressure is low, specifically the Ethiopian highlands, Rwanda, Burundi and to a lesser degree Nigeria. We therefore conclude that the key merit of agronomic fortification should rest with increasing agricultural productivity but that other types of interventions are more cost-effective in combatting malnutrition.

## Conceptual approach and methods

The analysis in this paper is based on the conceptual model described by Eq (1), in which we set to explain variation in four different health indicators (H), from factors $\vec{F}$ describing differences in soil quality and a set of controls ($\vec{C}$). Eq (1) is estimated for four different health indicators available in the Demographic and Health Survey [19] (details available in the data Section 3). These are child mortality, prevalence of underweight children, prevalence of child stunting and prevalence of child wasting. Factors $\vec{F}$ describing differences in soil data are based on the detailed grid level data on soil nutrients estimated by Hengl et al. [18]. We use factor analysis to reduce the dimensionality of this dataset. This is both a useful exercise in itself, providing insight into spatial correlation amongst soil nutrients, as well as a means to reduce potential problems of multi-collinearity in the estimation approach. The hypothesis is that increases in soil nutrient densities, i.e. increases in F, translate into a reduction in incidences of mortality, stunting, wasting or underweight. In other words, we expect negative estimates for β which signal an increase in health due to soils richer in nutrients. Finally, a set of controls $\vec{C}$ is included, using variables used in other recent econometric spatial analyses of African development. Details on all these variables is provided in the data section (Section 3).

$$H=\alpha +\beta \vec{F}+\gamma \vec{C}+\varepsilon$$ (1)

There could be two distinct, though partially overlapping, pathways of impact. First, richer soils signal a greater agricultural production potential. This is a logical analogy to the finding that fertilization with micronutrients has a positive effect on crop yields in SSA [16]. Or, if such nutrients are already naturally available in sufficient quantities this should also imply greater crop productivity. Then, all other things being equal, a greater production potential translates into greater income for local farmers. The relation between better soils, greater income and or reduced poverty has now been established in various studies across the developing world [20]. Such an income effect allows farmers to consume more, not necessarily own produce, and/or to smooth income fluctuations and consumption shortfalls better. Greater income allows for increased spending on health services, the purchase of more and food of better quality including more animal-based food products. In all these cases the actual consumption of proteins and micronutrients may increase, both of which lead to reductions in malnutrition [21].

That increases in (agricultural) income translate into better nutrition security may seem straightforward, but empirical assessments of such a relation are relatively scarce and difficult to interpret due to contemporaneously confounding effects such as institutional quality (e.g. [22]). One study [23] from Malawi did identify increases in nutrition resulting from the Malawi national fertilizer subsidy program. Since this program does not cover fertilizers enriched with micronutrients, this result primarily hints at improved nutrition through an income effect.

The second reason for a positive effect of soil nutrients on human health relates to increases in micronutrient availability in harvested product. Since richer soils translate into greater production, the amount of nutrients contained in harvested product increases as well. But, it is more difficult to predict whether or not the relative content of nutrients in harvested product changes. So-called yield dilution could occur. For instance, the widespread application of macronutrients through fertilizers has been cited as a reason for decreasing relative micronutrient content in food items [24]. That being said, the various agronomic field trials cited in Joy et al. [12] and Kihara et al. [16] do observe increases in nutrients stored in plant tissue as a result of agronomic fortification. Soils richer in soil nutrients could thus either translate into more production, or production with greater nutritive content, or both. Either way, the total volume of nutrients stored in harvested crop products is bound to increase, along with the harvested crop product itself. Furthermore, the share of Africa’s agricultural production that is exported is small, and most of the production is consumed at locations close to the farm [25, 26]. It is thus likely that the volume of nutrients stored in crops destined for local consumption, for feeding a given population in a grid cell, is greater in those regions with richer soils.

By estimating (1), we estimate a reduced form and assume that all relevant income and consumption effects, resulting from variation in soil nutrients, have evolved into an equilibrium effect captured by parameter β. Our estimation approach closely follows the approach used by Michalopoulos and Papaioannou [27], also including many of the same control variables. We estimate (1) using OLS in Stata and since the soil data is a cross-section so is our estimation approach. Observations are arranged at the lowest possible grid cell level, in our case grid cells at a resolution of 5km x 5km. We cluster standard errors twice, using the multiway clustering approach developed by Cameron et al. [28] (available through the *reghde* command available for Stata developed by Correia [29]) in order to account for unobserved spatial correlations. In each specification, we cluster at the country level and either at the regional level (for which most health data is provided) or at the level of precolonial institutions (also see data description).

Our estimates of (1) does not directly discriminate between either of two possible chains of causality. In line with virtually all other studies on malnutrition we can only rely on detailed data on the outcomes or symptoms of malnutrition, i.e., the anthropometric measurements, as spatially disaggregated food consumption surveys are mostly unavailable.

However, cross-terms between soil nutrients and the share of marketed produce could reveal whether a positive effect between soils and health is stronger for households (or regions) in which own consumption of agricultural produce is more common. This could suggest that the nutrient supply mechanism dominates. Unfortunately, continent-wide data on the share of marketed produce (and subsistence consumption) is unavailable and we resort to proxies for market development.

In the final step, we monetize the costs underlying the estimated marginal effect β, by calculating the underlying market value of an increase in soil micronutrients. In other words, how much would it cost to buy a specific volume of micronutrients on world markets, and using this volume to increase the density of soil nutrients in a particular grid cell? We use recent market prices from the London Metal Exchange to monetize these costs. Obviously, this calculation reflects a lower bound as it does not account for actual fertilizer product development, transportation and marketing costs.

We make this calculation for one combination of nutrients and health outcome only, namely for micronutrients (zinc, manganese and copper) in relation to child mortality. To be precise, we calculate the costs of one child death averted resulting from increasing the soil nutrient content of these elements. Since much of current research on malnutrition focusses on the role of zinc in relation to mortality and stunting, it provides ample cost-estimates to compare our figures with. We leave the monetization of costs and benefits of other elements, and their interpretation, to future research.

## Data

In order to estimate (1) several existing data sets have been adapted and merged into a Stata data file. This section describes key details of data sources and the adaptations carried out. Full detailed Stata scripts are provided in the appendix (S1 Dataset).

### Health data

We set to explain variation in four health indicators (Table 1) commonly used to assess the impact of malnutrition in Sub-Saharan Africa. For instance, in 2010, childhood underweight was responsible for around 19% of under-five deaths and Disability Adjusted Life Year (DALY) loss, zinc deficiencies for around 5% of under-five deaths and DALY loss and iron deficiencies for around 0.6% of child deaths and 6% of total DALY loss [3]. Indicators used in this analysis are those from the Demographic and Health survey [19]. Highly detailed maps revealing spatial variation in incidences of stunting and wasting (at 5km x 5km) across Africa have been published recently [30]. The estimation procedure used to generate these maps relies on covariates that are similar to the ones underlying the estimates of soil nutrient maps used in this study (see section 3.2). For this reason, these maps, and the underlying data, are unsuitable for use in our assessment for risk of identifying a spurious relationship. These indicators are provided at subnational level for all African countries. As the spatial resolution of our soil data is greater, we assume that the average health indicators in each grid cell are equal to the district averages. Table 1 provides definitions, means and standard deviations. It shows, for instance, that the average child mortality rate is 44, meaning that for every 1,000 children reaching their fifth birthday, 44 children have passed away.

**Table 1:** Health indicators used.

Indicators	Simple description	Exact definition	Mean	S.D.
H_1_	Child mortality: odds of dying too young	Mortality rate (out of 1000 children surviving their first birthday) of children between 1^st^ and 5^th^ birthday.	44.47	24.28
H_2_	Child stunting: height of children too low for age	% of children with height for age more than two standard deviations below WHO reference.	38.53	9.00
H_3_	Child wasting: weight of children too low for height	% of children with weight for height more than two standard deviations below WHO reference.	9.74	6.08
H_4_	Child underweight: weight of children too low for age	% of children with weight for age more than two standard deviations below WHO reference.	21.33	9.23

### Soil data

**Fig 1:**
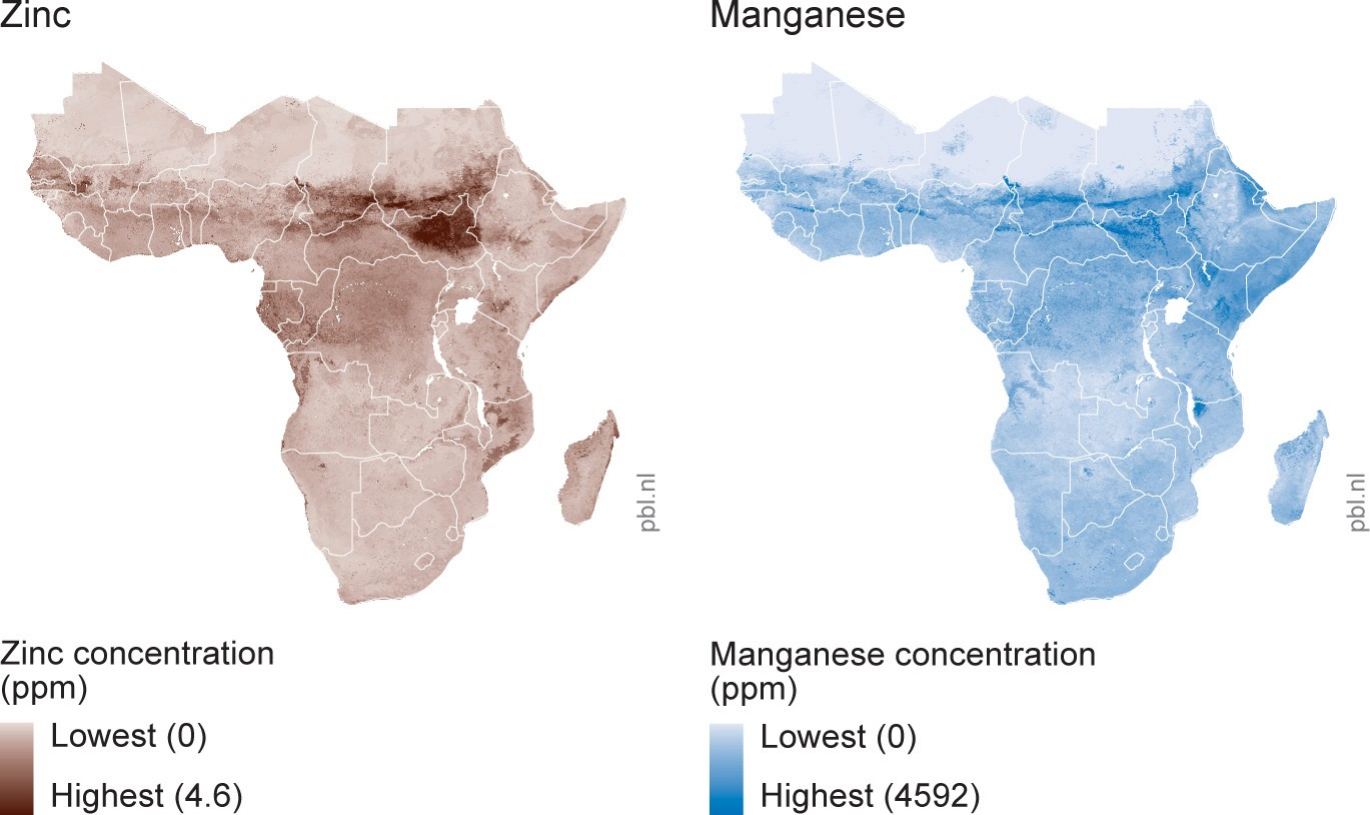
Estimated concentrations of zinc and manganese in soils across Sub-Saharan Africa. Data based on Hengl et al. [18]

The detailed spatial predictions of soil nutrient densities by Hengl et al. [18] form the basis of our analysis. Their approach is based on the compilation of a geo-referenced dataset of soil samples across Africa from the past 50 years, together comprising a dataset of around 55,000 unique point estimates. Hengl et al. [18] use machine learning to estimate variation in soil nutrient densities as a function of an array of geophysical/climatic variables. These estimates are subsequently used to predict soil nutrient densities in all grid cells for which no soil samples exist. Fig 1 shows such extrapolations for zinc and manganese concentrations.

Such predictions, for grid cells of 250m by 250m, form the basis for the soil factors $\vec{F}$ used in our analysis. We aggregate and average the soil data to grid cells of 5km by 5km as much of the other data used is not available on such a detailed scale. All data (Table 2) are an estimate of the nutrient content (macro- and micronutrients, as well as organic matter content) in the topsoil (30 cm) and are available for 813,704 point estimates across Sub-Saharan Africa [18]. The original data provides information on particles per million, which is converted to kilograms per hectare as discussed in Berkhout et al. [10] This data considers Sub-Saharan Africa as all land mass below a latitude of 28 degrees north. Hengl et al. [18] also provide estimates for phosphorus (P) and sulfur (S). As the fit of these estimates is very low (R^2^ of 0.11 and 0.10 respectively) these elements are not included in this analysis.

**Table 2:** Key characteristics of soil data used.

Variable	Mean (kg/ha in 30cm topsoil)	Std. Dev. (kg/ha in 30cm topsoil)
Boron (B)	0.29	.21
Calcium (Ca)	1041.29	842.46
Copper (Cu)	1.06	.86
Iron (Fe)	39.44	27.07
Potassium (K)	69.96	51.72
Magnesium (Mg)	166.06	136.09
Manganese (Mn)	41.18	22.56
Nitrogen (N)	315.00	162.88
Organic Matter Content (OMC)	4.64	3.39
Zinc (Zn)	1.58	1.18

Altogether 10 variables are thus available for inclusion in (1). We use factor analysis to reduce the dimensionality of this dataset. If one or more variables co-vary strongly, the estimation of (1) is affected by multi-collinearity. In addition, factor analysis is likely to reduce the impact of measurement error underlying the variables in Table 2. Moreover, an understanding of which soil nutrients co-vary is insightful information in itself. In fact, strong communal variation between certain soil variables may stem from the fact that certain geological or soil formation processes affect soil nutrient densities similarly.

Tables 3 and 4 display the outcome of a standard factor analysis, carried out in Stata using the commands *factor* and *rotate* with default options. Commands of the full data adaptation and estimation procedures are found in the Stata do-files available with this publication. The goal is to reduce the 10 soil nutrient variables to a smaller set of variables that still account for most of the variation present in the original data (e.g. [31]). Such a reduction is possible if, and only if, two or more variables are strongly correlated. In such cases these correlated variables can be proxied by a single new variable (factor). The first step is the initial factor extraction (Table 3) and the second step involves a rotation of these extracted factors that allows for clearer interpretation (Table 4). The rotated factors displayed in Table 4 are the ones included in our analysis.

**Table 3:** Exploratory factor loadings.

Variable	Factor1	Factor2	Factor3	Factor4	Uniqueness
Boron (B)	0.4318	0.0928	0.3736	0.4125	0.4953
Calcium (Ca)	-0.0505	**0.8511**	-0.3510	0.0815	0.1431
Copper (Cu)	**0.8337**	0.0314	0.1021	0.0894	0.2856
Iron (Fe)	0.2387	-0.6075	0.5614	0.0626	0.2549
Potassium (K)	0.2260	0.5141	0.1652	0.5745	0.3273
Magnesium (Mg)	0.2765	**0.7749**	-0.1161	0.1405	0.2898
Manganese (Mn)	**0.7647**	0.1015	0.1193	0.3781	0.2478
Nitrogen (N)	0.1758	-0.1981	**0.7975**	0.1497	0.2715
Organic Matter Content (OMC)	0.1245	-0.3049	**0.7808**	0.0199	0.2816
Zinc (Zn)	**0.8089**	-0.0140	0.2026	-0.0649	0.3002

Table displays factor loadings resulting from a factor analysis carried out on the soil nutrient variables (Table 2).

**Table 4:** Predicted factor loadings.

Variable	Factor1	Factor2	Factor3
Boron (B)	0.02394	0.01475	0.05746
Calcium (Ca)	-0.06596	**0.62579**	0.09460
Copper (Cu)	**0.36668**	-0.00885	-0.08382
Iron (Fe)	0.04641	-0.15850	0.15683
Potassium (K)	-0.07706	0.06731	0.07392
Magnesium (Mg)	0.09113	**0.28120**	0.10559
Manganese (Mn)	**0.29581**	-0.06854	-0.14837
Nitrogen (N)	-0.09126	0.16747	**0.45675**
Organic Matter Content (OMC)	-0.05701	0.08977	**0.42514**
Zinc (Zn)	**0.35496**	0.01262	0.01109

Table displays a Varimax rotation of the factors extracted in Table 3

Table 3 displays the first four factors extracted, those with positive eigenvalues. The eigenvalues are a reflection of the accuracy by which the factors account for the original underlying variation. As a rule-of-thumb (the so-called Kaiser criterion) [31] typically only factors with eigenvalues greater than one are used in further analysis. In Table 3, this holds for the first two extracted factors only (3.41 and 2.72 respectively). We do, however, also include the third factor (despite the lower eigenvalue of 0.76), primarily as it is the only factor that appears to capture variation in nitrogen and organic matter well (two important and often used indicators of soil fertility and quality).

Table 3 displays the exploratory factor loadings, which are the correlations between the original variables and the newly constructed factors. The strongest variations (>0.75) are highlighted in bold. Thus, factor 1 strongly captures communal variation in three micronutrients (copper, manganese and zinc). The second captures variation of macronutrients (calcium and magnesium) and the third factor captures communal variation between macronutrient nitrogen and soil organic matter content. The last column in Table 3 displays so-called uniqueness. This is a measure of the variance of the particular variable that is not captured in underlying communal variance. The higher it is, the more unique a variable is and the less of its variance co-moves with the variance in other variables. With the possible exception of boron, most of these uniqueness scores are fairly low, indeed suggesting that much of the variance is communal. We subsequently carry out a rotation (Varimax rotation) for these three factors allowing for easier interpretation (Table 4).

Table 4 lists newly constructed soil factors that are a linear combination of the original, but normalized, data from Table 2. It thereby maintains the strength of the factor loadings as presented in Table 3. For instance, factor 1 is constructed as follows:

$$Factor1=f_{B}*\bar{B}+f_{ca}*\bar{Ca}+f_{cu}*\bar{Cu}+\;\ldots +f_{Zn}*\bar{Zn}$$ (2)

Hereby, $\bar{B}$, $\bar{Ca}$ and $\bar{Cu}$ (and so on and so forth) reflect normalized variables or z-scores. The factor loadings $f_{B}$, $f_{ca},\;f_{cu}$ in (1) are those as presented in Table 4. Thus, the first factor is mostly a weighted mean of micronutrients based on the standardized variables of copper (0.36668), manganese (0.29581) and zinc (0.35496). Variation from the remaining soil variables only contributes very modestly to factor 1. The second factor captures variations in calcium and magnesium densities and the third factor is strongly associated with nitrogen soil densities and organic matter content. The strong communal variation between some variables, like zinc, manganese and copper, also suggests that it is fairly difficult to isolate causal effects from one of these elements separately. Or, there is too little idiosyncratic variation in one element, while holding the others constant, to tease out its impact on health outcomes. We subsequently use these factors to estimate the effects of soil differences on health outcomes in the next sections.

### Other control variables

We further include a set of controls $\vec{C}$, largely in line with other recent spatial analyses of African development (e.g. [27]), to take account of various other variables that may explain differences in health outcomes. An overview of variables used and their data source is presented in Table 5. These variables describe differences in economic development, or potential, and as discussed in detail below may further shape nutrient availability and consumption.

**Table 5:** List of all control variables included.

Variable	Name in analysis	Unit / values	Description and data source
Night time luminosity	*nightTime*	0–63	NOAA—National Centers for Environmental Information [32]. Data for 2013 is used. Greater values signal greater night time luminosity.
Night time luminosity	*lightDum*	Dummy: 1 if grid cell is lit; 0 otherwise	Own calculations using data from NOAA—National Centers for Environmental Information [32]).
Population density (log)	*ln_pop*	Log(# per km^2^)	Center for International Earth Science Information Network—CIESIN—Columbia University [33]. Data for 2015 used. Accessed 3-06-2017
Institutional Hierarchy	*hierarchy*	0–4	As developed by Murdock [34] using variable code v33 in the Ethnolinguistic Atlas. It describes the number of political jurisdictions above the local level for each ethnicity. A zero indicates stateless societies. A score of 1 indicates petty chiefdoms. A score of two indicates paramount chiefdoms, 3 and 4 indicate groups that were part of larger states.
Malaria stability index	*malaria*	0–39	Index based as from Kiszewski et al. [35]. Greater values signal greater malaria pressure.
Location of water bodies	*waterDum*	Dummy: 1 if water body covers grid cell; 0 otherwise	Calculated in ArcGIS using DCW waterbodies
Locations of diamond mines	*diamDum*	Dummy: 1 if diamond mine is located in grid cell; 0 otherwise	Data on locations of diamond as developed by Gilmore et al. [36].
Location of petroleum fields	*petroDum*	Dummy: 1 if petroleum field is located in grid cell; 0 otherwise	Data on locations of petroleum fields as developed by Lujala et al. [37].
Distance to country capital (log)	*capitalDis*	Log(m)	Distance of grid cell centroid to country capital, computed in ArcGIS using function *near*
Distance to nearest country border (log)	*borderDis*	Log(m)	Distance of grid cell centroid to nearest country border, computed in ArcGIS using function *near*.
Distance to nearest sea coast (log)	*coastDis*	Log(m)	Distance of pixel to nearest sea coast, computed in ArcGIS using function *near*.
Dummy for country being landlocked	*Landlocked*	Dummy: 1 if country has no own sea coast; 0 otherwise	Own calculations

As our exposition in Section 2 on the possible causal mechanisms pointed out, it is likely that differences in income affect health outcomes. We proxy levels of local economic development by including night time luminosity (*nightTime* and *lightDum*) as control variables. This variable, measured by satellites, is considered a robust proxy for local economic development (e.g. [38–40]). However, differences in luminosity might also arise from the underlying (pitch black) larger water bodies, at which obviously no measurable economic activity takes place. Moreover, the presence of highly lit petroleum fields and diamond mines may overstate the actual level of economic activity. We include three geographic control dummies to capture such possible effects (*waterDum*, *diamDum*, *petroDum*). Next, we control for differences in population density (*ln_pop*) since a greater number of people in a grid cell, all things being equal, lowers per capita availability of nutrients from harvested produce. In addition, we include a variable that captures differences in pre-colonial institutional organisation (*hierarchy*). This follows the revealed impact of pre-institutions on current institutions and current economic activity [27] and more broadly the assumed role of institutional quality in explaining health outcomes (e.g. [22, 41].

In addition, we include the malaria stability index developed by Kiszewski et al. [35]. Malaria is one of several parasitic diseases that are known to interact with malnutrition. In addition, incidences of intestinal parasites, diarrhea, pneumonia, measles and AIDS are known to impair nutrient intake leading to increased malnutrition [42]. But, to complicate matters, in some instances malnutrition may also increase the likelihood of contracting these diseases. The direct availability of the spatially disaggregated malaria indicator is one motivation for its inclusion, next to specificities on its interaction with malnutrition. Specifically, a hypothesis is that iron deficiency and resulting anaemia could actually aid with fighting off malaria infections as anaemia makes it more difficult for malaria parasites to propagate (e.g. [43]). In other words, reductions in iron deficiency in areas, where malaria proliferates, could actually worsen health outcomes. We include interaction effects between malaria and our soil nutrient factors to account for such possible effects.

Last, we include a number of location controls: distances from the centroid of each grid cell to the national capital, closest country border and distance to the closest sea coast (*capitalDis*, *borderDis*, *coastDis*). These variables account for differences in transport costs of commodities, and thus in net income and consumer prices, as well as the declines in the intensity of policy implementation as a function of the distance to the capital (see e.g. [44]). In addition, we include a dummy (*Landlocked*) signalling countries that do not have own sea coast. In these countries, trade also involves the crossing of a border with a neighbouring country, further adding transaction and transport costs.

In Section 2, we discussed the existence of two potentially different mechanisms that cause soil nutrient densities to affect health outcomes, one running through a change in income, the other through a direct increase in the local supply of nutrients in crops available for consumption. In the analysis, we investigate whether any of these mechanisms may dominate. For instance, if the share of consumption of own agricultural produce was known, this analysis is relatively straightforward. If the effect of soils on health is larger for households selling more own production, then the income effect dominates (and vice versa). Unfortunately, data on the marketed share of own production is not available for the full continent. Instead, we consider night time lighting and distances to capital, border and coast as proxies for market development and the importance of subsistence consumption, the latter being greater in dark and isolated areas. We estimate several models in which interactions of these variables with nutrients are estimated in order to explore the dominance of either mechanisms.

Most of the control variables are in line with those used by Michalopoulos and Papaioannou [27], the key exception being the land suitability index. We hypothesize that the rich soil data in this study, and subsequent factors delineated in the previous section, are both a better proxy for land suitability and are more relevant to examining the effect on health and nutrition-related dependent variables. Moreover, the soil data itself is a function of various climatic and geophysical data such as temperature and rainfall differences (see [18] for more details). This does not invalidate our approach, but rather adds a logical layer of causality to the posited chain of causality. Thus, climatic and geophysical variables shape local soil nutrient contents, which determine agricultural potential and production, which in turn defines nutrient availability for local consumption and eventually determines nutrition and health outcomes.

Arguably, in a cross-section analysis as ours concerns on endogeneity of one or multiple variables can never be fully eliminated. Ideally, instrumental variables capturing deeper geological processes are available to instrument for current nutrient densities. Unfortunately, the original analysis [18] provides no obvious and strong instruments. That said, by reasoning, endogeneity may only bias our estimation results to a very limited extent, given the structure of (1) as well as the variables included above. First, it is unlikely that current health outcomes have a direct effect on soil quality, especially considering the fact that much of the underlying soil data has been collected over the past 50 years. A similar argument holds for the relation between pre-colonial institutional quality and health. All other variables, bar night time luminosity, are strictly exogenous. Health outcomes may, however, distinctively shape local economic activity and thus night time luminosity. For this reason, we use night time luminosity data of 2013, while the most recent (2013) health data is used. Unfortunately, for a few countries the most recent health data is from surveys before 2013. As a final means to test the stability of our findings we therefore estimate (1) with the controls on night time lighting and population density omitted, variables that potentially harbour some endogenous interaction with nutrient densities.

## Impact of soil nutrients on health

We estimate model (1) using the data and the methodology as outlined in the previous sections. Table 6 displays model estimates for child mortality. The differences between the models are explained in the footnotes and relate to differences in clustering variables, the inclusion of night time lighting as a continuous variable, and the inclusion of interaction effects between soil quality variables and malaria pressure.

**Table 6:** Regression results explaining variation in child mortality.

VARIABLES	(a)	(b)	(c)	(d)	(e)	(f)	(g)
Cu–Mn–Zn	-2.129***	-8.275***	-8.275**	-8.275**	-7.756*	-7.411*	-6.350*
	(0.0604)	(0.105)	(3.998)	(4.066)	(3.872)	(4.359)	(3.335)
Cu–Mn–Zn * Malaria Index		0.465***	0.465*	0.465*	0.436*	0.412	0.281
		(0.00565)	(0.268)	(0.259)	(0.249)	(0.261)	(0.225)
Ca–Mg	1.142***	1.695***	1.695	1.695	1.724	2.246	3.443
	(0.0501)	(0.0750)	(4.110)	(4.014)	(3.912)	(4.067)	(4.002)
Ca–Mg * Malaria Index		-0.181***	-0.181	-0.181	-0.188	-0.153	-0.215
		(0.00556)	(0.314)	(0.313)	(0.309)	(0.323)	(0.338)
N–OMC	-4.110***	-4.484***	-4.484	-4.484	-4.560	-4.163	-4.579*
	(0.0423)	(0.0612)	(2.981)	(2.910)	(2.778)	(3.047)	(2.667)
N–OMC * Malaria Index		0.0658***	0.0658	0.0658	0.0615	0.0128	0.100
		(0.00462)	(0.287)	(0.275)	(0.263)	(0.283)	(0.252)
Institutional hierarchy	-0.786***	-0.979***	-0.979	-0.979	-0.914	-1.735	-0.601
	(0.0361)	(0.0366)	(1.775)	(1.661)	(1.654)	(1.659)	(1.456)
Malaria Index	0.824***	0.775***	0.775**	0.775**	0.748**	0.738**	0.785**
	(0.00467)	(0.00491)	(0.329)	(0.334)	(0.328)	(0.345)	(0.320)
Population density (logs)	2.443***	2.877***	2.877***	2.877***	3.163***	3.384***	3.258***
	(0.0221)	(0.0233)	(0.890)	(0.914)	(0.897)	(0.989)	(0.834)
Night time luminosity (dummy)					-14.06***	-14.20***	-11.18***
					(2.308)	(2.378)	(2.406)
Water body (dummy)						-4.144	
						(3.695)	
Petroleum site (dummy)						-5.369	
						(4.800)	
Diamond mine (dummy)						6.184	
						(5.578)	
Night time luminosity (level)	-1.072***	-1.067***	-1.067***	-1.067***			
	(0.0150)	(0.0150)	(0.194)	(0.193)			
Distance to capital (log)							1.186
							(1.423)
Distance to coast (log)							3.603***
							(1.103)
Distance to border (log)							-1.097
							(0.746)
Landlocked (dummy)							-1.781
							(4.948)
Constant	31.95***	29.71***					
	(0.0753)	(0.0816)					
Observations	359,019	359,019	359,019	359,019	359,019	324,626	358,978
R-squared	0.201	0.218	0.218	0.218	0.231	0.237	0.259

Standard errors in parentheses:

*** p<0.01,

** p<0.05,

* p<0.1.

Dependent variable is Child Mortality in all specifications. Models differ in the following ways:

(a): no clustering on standard errors, night time as continuous variable

(b): as (a), with interaction effects between malaria and soil nutrient factors

(c): as (b) with double clustering at country and precolonial institutions level, night time as continuous variable

(d): as (b) with double clustering at country and regional level, night time as continuous variable

(e): as (d), with night time as dummy (0: dark cells; 1 lighting)

(f): as (e), with geographic controls

(g): as (e), with location controls

Altogether the differences between models with either single clustering (model a and b) or double clustering (models c-g) of standard errors are small. Much of the unobserved correlation between standard errors is observed at the country level, and to a much smaller degree at the pre-colonial institutional or regional level. Moreover, virtually no differences occur when double clustering either at the level of countries and pre-colonial institution, or at the level of countries and regions. Given these minor differences we continue using double clustering at country and region-level.

The estimates explain a considerable amount of the variance in current child mortality. Considering the statistically significant variables, variation in soil quality, malaria pressure, night time lighting and distance to coast and capital thus explain up to 26% of child mortality across Sub-Saharan Africa. The effects of soil quality are apparent and significant. Consider, for instance, the effect of the micronutrient factor 1. A unit increase in this factor reduces child mortality in the range of 2 to 8 per mille points, thus reducing child mortality from around 44 deaths per 1,000 to a range of 36–42 deaths per 1,000. That said, the precise effects are somewhat obscured by the significant interaction effects between malaria and soil nutrients. We calculate the precise distribution of marginal effects later on.

The effects of the various control variables as documented in Table 6 are in line with a priori expectations. Increasing malaria pressure leads to increases in child mortality. Greater population densities lead to increases in mortality, possibly due to decreasing per capita nutrient availability. Child mortality is considerably lower in (more urbanized) areas with greater night time luminosity, possibly due an income effect, the availability of more and better health services or the availability of more diverse diets. Distance to the coast increases mortality rates, likely reflecting an increase in transport costs. Such significant effects of soil factors on health outcomes are not confined to child mortality only and are observed in relation to child stunting, wasting and underweight as well.

Table 7 shows model estimates for the four health indicators together, each estimated similar to model (g) in Table 6. The signs of the control variables are mostly similar to the results for child mortality even though the effect of the control variables is not always significantly different from zero. For instance, there appears a fairly robust effect of the night time luminosity (i.e. greater economic activity and income) on the different health outcomes. Again, increasing distances to the coast seem to increase mortality rates as well as distances to the capital. The latter likely reflects declining government spheres of influence, for instance, with respect to health policies including local availability of medical care. On the other hand, the effect of precolonial institutions (*hierarchy*) does not appear statistically significant in any of the models estimated.

**Table 7:** Regression results explaining differences in child mortality, child stunting, child wasting and child underweight.

	(1)	(2)	(3)	(4)
VARIABLES	Child mortality	Child stunting	Child wasting	Child underweight
Cu–Mn–Zn	-6.350*	-3.235*	-3.028***	-3.635*
	(3.335)	(1.727)	(1.076)	(1.967)
Cu–Mn–Zn * Malaria Index	0.281	0.183	0.0825**	0.139
	(0.225)	(0.111)	(0.0404)	(0.0907)
Ca–Mg	3.443	-2.559*	3.297***	4.566***
	(4.002)	(1.334)	(0.671)	(1.563)
Ca–Mg * Malaria Index	-0.215	0.162	-0.0859	-0.0811
	(0.338)	(0.0990)	(0.0767)	(0.138)
N–OMC	-4.579*	1.910**	-2.239***	-1.127*
	(2.667)	(0.839)	(0.567)	(0.662)
N–OMC * Malaria Index	0.100	-0.152	0.0542**	-0.0216
	(0.252)	(0.0922)	(0.0251)	(0.0716)
Institutional hierarchy	-0.601	0.0248	-0.429	-0.791
	(1.456)	(0.607)	(0.564)	(0.956)
Malaria Index	0.785**	-0.121	0.203***	0.222**
	(0.320)	(0.0774)	(0.0634)	(0.0929)
Population density (logs)	3.258***	1.166***	0.480	1.076**
	(0.834)	(0.298)	(0.291)	(0.410)
Night time luminosity (dummy)	-11.18***	-6.069***	-0.462	-3.702***
	(2.406)	(1.082)	(0.507)	(1.020)
Distance to capital (log)	1.186	3.014***	0.870***	1.834***
	(1.423)	(0.551)	(0.298)	(0.549)
Distance to coast (log)	3.603***	0.600	0.673**	1.176*
	(1.103)	(0.808)	(0.324)	(0.697)
Distance to border (log)	-1.097	0.301	0.0113	0.0488
	(0.746)	(0.265)	(0.147)	(0.240)
Landlocked (dummy)	-1.781	4.170***	1.552	3.125
	(4.948)	(1.489)	(0.997)	(2.006)
Observations	358,978	311,285	298,950	298,950
R-squared	0.259	0.353	0.372	0.311

Robust standard errors in parentheses,

*** p<0.01,

** p<0.05,

* p<0.1

We estimate the effects of multiple soil nutrients on multiple health outcomes, which could lend suggestion of being a form of specification mining. Given the number of t-tests in all of these regressions the likelihood of a Type 1 error occurring may indeed be fairly large. Options to control for such compounding errors exists by means of multiple hypothesis testing, most notably through the Bonferroni and derived corrections. But the usefulness of applying such corrections in more exploratory studies as ours remains under debate (e.g. [45]). We therefore choose not to apply any such corrections, a choice further strengthened by the observation that our findings (of a link between soils and health) do not hinge on a single significant coefficient, but rather on a more consistent finding of multiple significant effects across the different health outcomes.

We subsequently explored whether or not the data provides insights into either of the mechanisms driving this effect between soils and health. We ran 48 additional regressions with interactions between each soil nutrient factor and night time lighting, or the three different geographical distance variables. Detailed results are available upon request. Such interactions with either the micronutrient factor, or with the nitrogen and organic matter factor are rarely significant. In few cases that they are, it suggests that the relation between soils and health is smaller in isolated areas, lending credence to an income effect as the driver of the effect on health. Yet, interactions with the calcium and magnesium factor point to the exact opposite. In fact, various interactions turn out significant (across the different health outcomes, for night time lighting as well as the geographical controls). Given these opposite findings a verdict on a dominating mechanism remains inconclusive and requires additional research, for instance, by using experimental approaches stratified across settings with different subsistence levels.

Two further methodological considerations are appropriate here. First, we noted that population density and night time lighting may be considered bad controls as these may be intricately linked with soil nutrients. We therefore investigated the stability of the estimates by omitting these potentially “bad” controls from (1). The results are provided in the appendix (S1 Table) but are largely similar to those reported in Table 7. The key difference being that micronutrients no longer explain child mortality. Second, our dataset is considerably large (around and over 300,000 observations) which is driven by the richness of the underlying soil dataset. Tables 6 and 7 reflect an analysis in which we disaggregated, or rather transposed, the observed health outcomes of a specific district to the unobserved outcomes in each location within that district. To our knowledge there is no objection on econometric grounds to the approach followed, except for the fact that measurement errors in child health outcomes in a district now carry over to all observations in that district, making the followed clustering approach across subnational regions or countries imperative. That said, increasing the number of observations in this way may somewhat artificially raise the odds of finding significant effects. Therefore, as a robustness check, we estimated (1) for all health outcomes with all variables aggregated (averaged) to the district level at which health outcomes were measured. The results are presented in the appendix (S2 Table), showing that most of the key findings remain (with respect to significance levels and size of coefficients) in such a more parsimonious data set.

**Fig 2:**
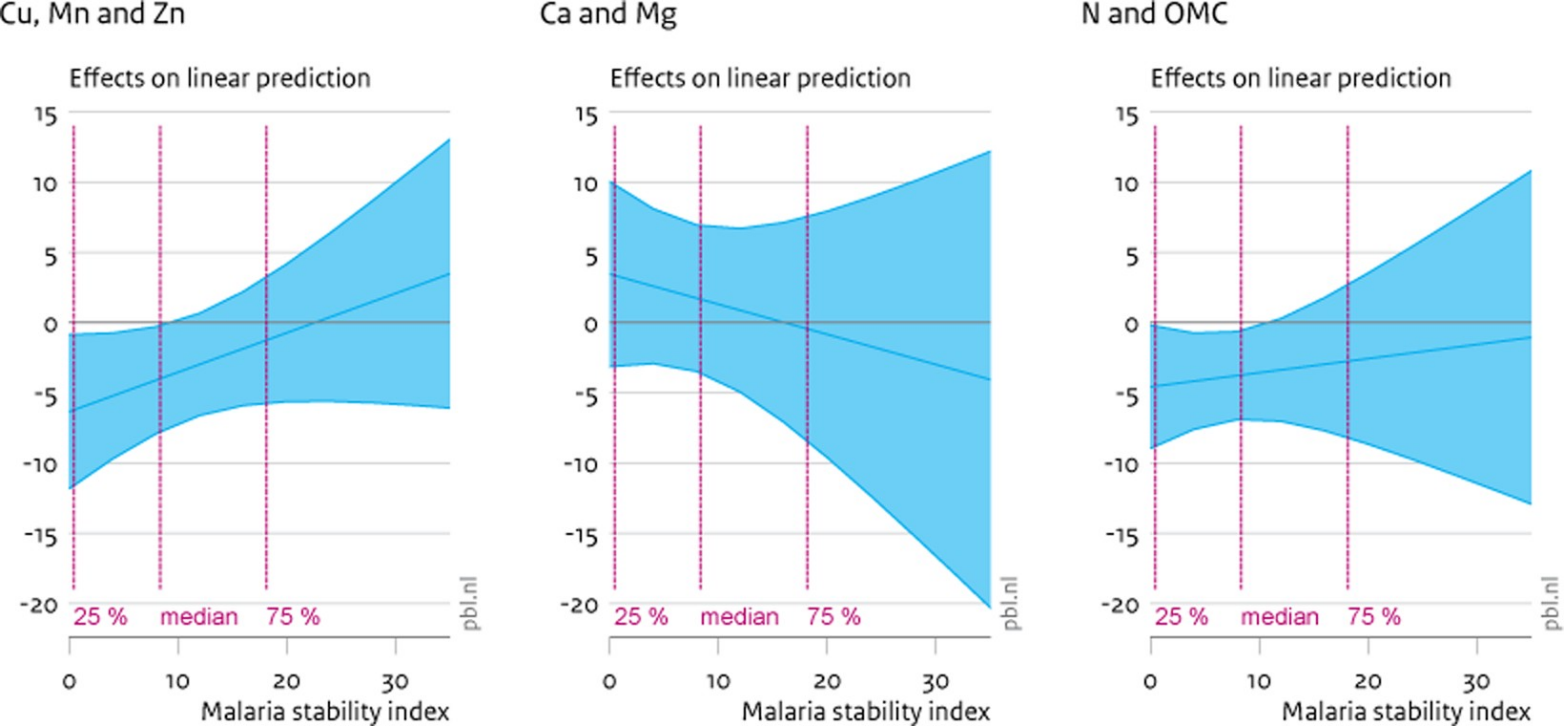
Marginal effects (90% CI) of soil nutrient increase on child mortality. The graphs display 90% confidence intervals of the marginal effects of greater soil nutrient densities on child mortality. Panel a displays the effects of factor 1 (Cu, Mn and Zn), panel b of factor 2 (Ca and Mg) and panel c of factor 3 (N and OMC).

**Fig 3:**
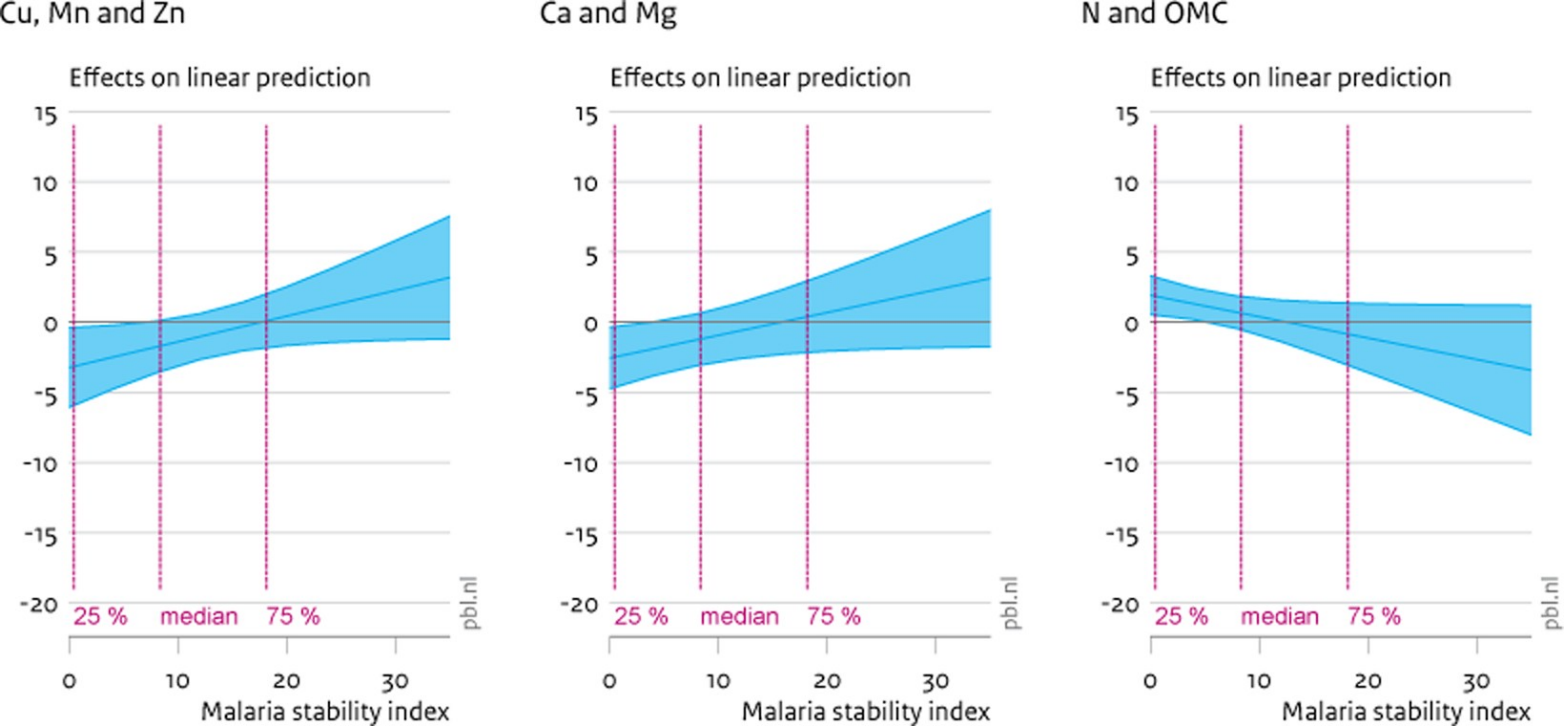
Marginal effects (90% CI) of soil nutrient increase on child stunting. The graphs display 90% confidence intervals of the marginal effects of greater soil nutrient densities on child stunting. Panel a displays the effects of factor 1 (Cu, Mn and Zn), panel b of factor 2 (Ca and Mg) and panel c of factor 3 (N and OMC).

**Fig 4:**
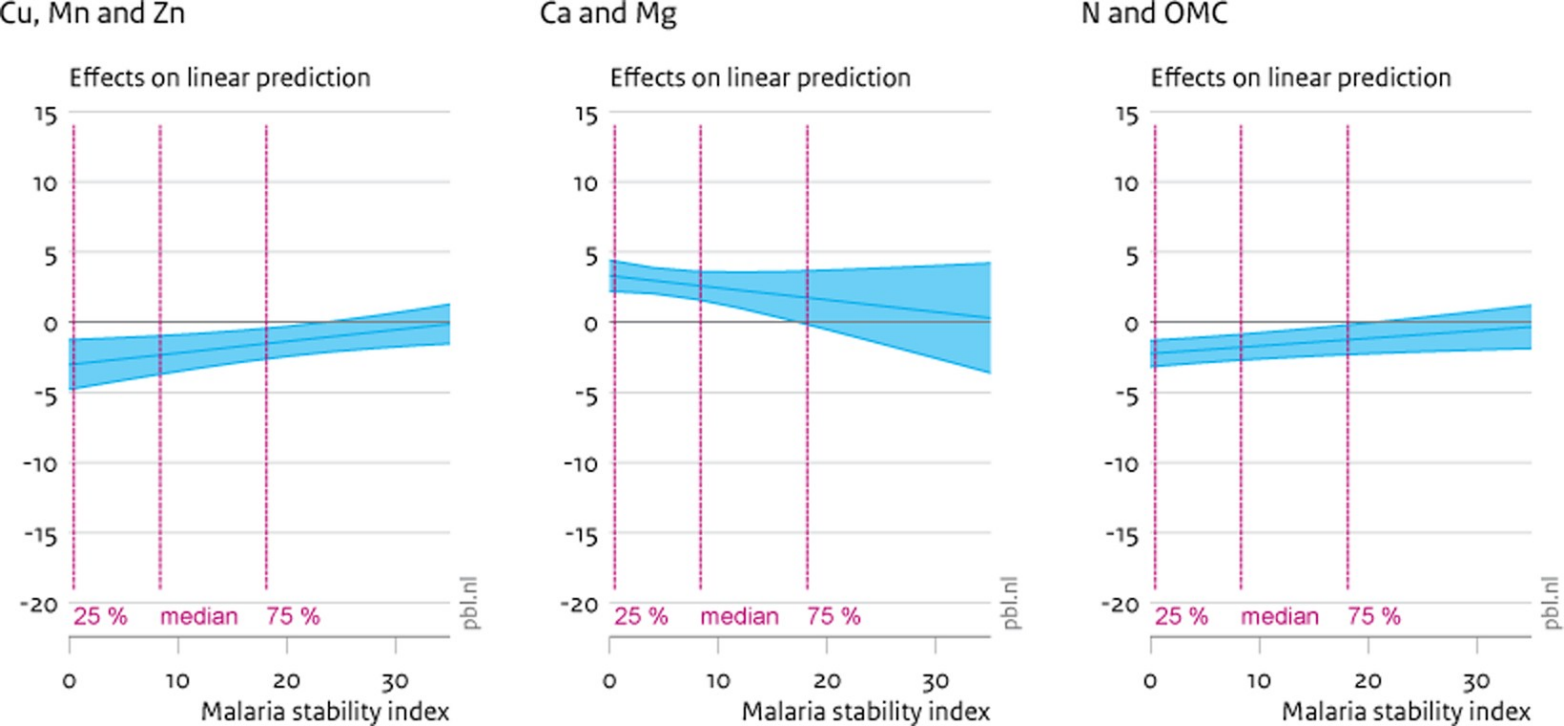
Marginal effects (90% CI) of soil nutrient increase on child wasting. The graphs display 90% confidence intervals of the marginal effects of greater soil nutrient densities on child wasting. Panel a displays the effects of factor 1 (Cu, Mn and Zn), panel b of factor 2 (Ca and Mg) and panel c of factor 3 (N and OMC).

**Fig 5:**
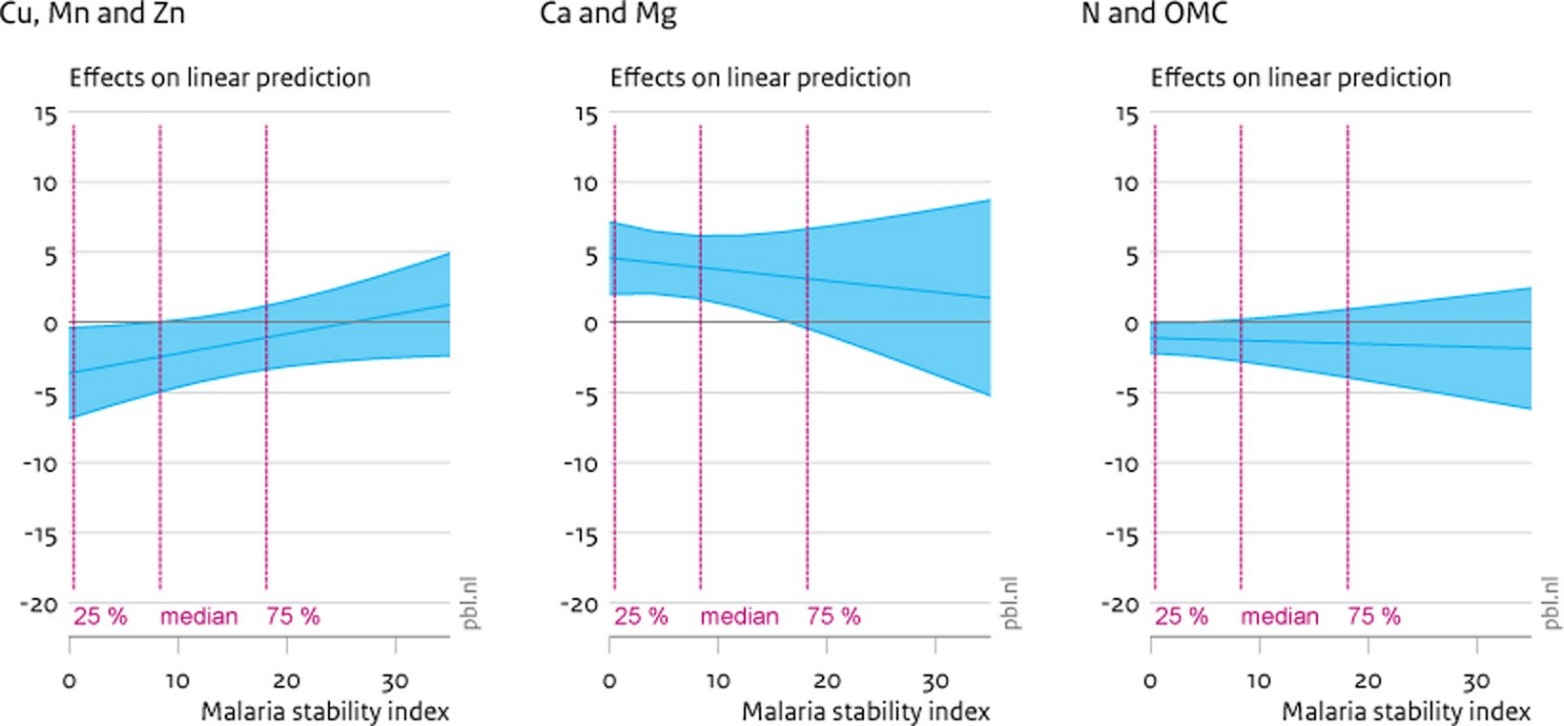
Marginal effects (90% CI) of soil nutrient increase on child underweight. The graphs display 90% confidence intervals of the marginal effects of greater soil nutrient densities on child underweight. Panel a displays the effects of factor 1 (Cu, Mn and Zn), panel b of factor 2 (Ca and Mg) and panel c of factor 3 (N and OMC).

Because of the significant interaction effects between the malaria stability index and the soil factors, the marginal effects vary for different values of the malaria stability index. We therefore calculate the precise marginal effects of soil factor increases, conditional on different values of the malaria stability index. These calculations are provided in Table 8, as well as displayed in Figs 2–5. Table 8 provides the approximate marginal effects at the 25%, 50% and 75% percentile of the malaria stability index, which corresponds to actual values of this index of around 0, 8 and 16 respectively. Figs 2–5 show 95% confidence intervals of the marginal effects of additional soil nutrients on child mortality, stunting, wasting and underweight. The x-axis reflects the malaria stability index and the vertical lines partition the data on malaria stability index into four quartiles.

**Table 8:** Marginal effects of soil factors on child mortality, stunting, wasting and underweight.

		(1)	(2)	(3)	(4)
VARIABLES	Malaria distribution at (approx. percentile)	Child Mortality	Child Stunting	Child Wasting	Child Underweight
**Cu–Mn–Zn**	0 (25%)	-6.350*	-3.235*	-3.028***	-3.635*
		(3.335)	(1.727)	(1.076)	(1.967)
	8 (50%)	-4.105*	-1.771	-2.368***	-2.520*
		(2.314)	(1.121)	(0.840)	(1.524)
	16 (75%)	-1.861	-0.307	-1.708**	-1.404
		(2.464)	(1.051)	(0.680)	(1.353)
**Ca–Mg**	0 (25%)	3.443	-2.559*	3.297***	4.566***
		(4.002)	(1.334)	(0.671)	(1.563)
	8 (50%)	1.727	-1.262	2.610***	3.916***
		(3.170)	(1.119)	(0.605)	(1.355)
	16 (75%)	0.0103	0.0344	1.923*	3.267*
		(4.320)	(1.406)	(1.017)	(1.919)
**N–OMC**	0 (25%)	-4.579*	1.910**	-2.239***	-1.127*
		(2.667)	(0.839)	(0.567)	(0.662)
	8 (50%)	-3.776**	0.691	-1.805***	-1.300
		(1.904)	(0.705)	(0.556)	(0.888)
	16 (75%)	-3.776**	-0.527	-1.372**	-1.473
		(1.904)	(1.174)	(0.615)	(1.341)

Robust standard errors in parentheses,

*** p<0.01,

** p<0.05,

* p<0.1

Fig 2 shows the impact of higher nutrient densities on incidences of child mortality. In Fig 2, panel a and c show that for locations with a malaria stability index below the median greater densities of soil micronutrients (Cu-Mn-Zn), as well as greater density of N and organic matter, lead to significant (at 95%) reductions in child mortality. These reductions are substantial, as also shown in Table 8. For instance, at the 25% percentile and at the median (50% percentile), the expected marginal effect of a unit increase in the first soil factor (capturing variation in Cu, Mn and Zn) leads to a reduction of 6.3 and 4.1 per mille points in child mortality respectively. However, the reductions in mortality, due to increasing densities in soil micronutrients dissipate when the malaria pressure increases. In the next section, we further discuss how such unit increases in the soil factors translate to actual differences in soil nutrient densities.

The panels in Fig 3 are similar in set-up to Fig 2, but now quantify the marginal effects of increased soil quality on child stunting. The first panel illustrates that, again, micronutrients have a significant impact on rates of child stunting (i.e. height of children too low for age). There is also a significant effect of the second factor reflecting densities of calcium and magnesium. For this factor, at rates of malaria pressure below the median, there appears a significant reduction of more calcium and magnesium on stunting. This is not unsurprising as higher intake of calcium has been associated with increases in skeletal growth and increased bone mass, leading to increases in height (e.g. [46]). This effect dissipates for higher rates of malaria pressure, where no significant effects of any soil factor on child stunting remains. Finally, at low levels of malaria intensity only, greater nitrogen densities and soil organic matter appear to increases levels of child stunting. While somewhat puzzling at first, the role of nitrogen and organic matter in child stunting is the precise inverse of the role in explaining child wasting (see Fig 4). More generally, nitrogen and organic matter, and to some degree micronutrients, are primarily weight enhancing, while calcium and magnesium are height-increasing.

Indeed, a similar effect of calcium and magnesium can be observed when assessing the marginal effects on child wasting (i.e. child weight too low for child height) (Fig 4). Here, the effect of calcium and magnesium is positive, i.e., it actually increases rates of child wasting. This effect is a different side of the same coin. When calcium and magnesium increase height, it will simultaneously increase rates of wasting, all else being equal. On the other hand, the other two factors seem to counter these effects. Both increases in micronutrients as well as nitrogen and organic matter content lead to considerable reductions in child wasting for rates of malaria pressure up to the 75% percentile. This suggests more of these elements in soils lead to intake of more, and more nutritious, food, in turn increasing weight. Again, at higher rates of malaria pressure no significant effects of soil nutrients remain.

Finally, Fig 5 documents the effect of changes in soil nutrients on incidences of child underweight. Here, the effect of micronutrients (Mn, Zn and Cu) mirrors the effects on child mortality. For low instances of the malaria stability index, additional micronutrients appear to reduce incidences of child underweight. There is a weak increasing effect, at low levels of malaria pressure, of calcium and magnesium on child underweight and no significant effect of nitrogen and organic matter.

## Costs and benefits of increasing soil nutrient content

The previous section has provided detailed estimates of the, statistically significant, spatial relation between local soil nutrient densities and local health outcomes. We argue that in the absence of field trials assessing the impact of enriched fertilizers on human health, the spatial relationship between soils and health elaborated above provides a first order indication -and currently best available empirical alternative- of the potential costs and benefits of using agronomic fortification to combat malnutrition. Given the strong focus in the scientific literature on the role of micronutrients, particularly zinc, in alleviating malnutrition we focus on the effects of the first soil factor used in the previous section (including zinc, copper and manganese). We assess the costs of enriching soils with these elements and contrast these with the benefits of reductions of child mortality.

We first establish the costs of increasing the densities of soil nutrients. This calculation is relatively straightforward by considering the definition of the factors as explained in Section 3.2. Specifically, a unit increase of factor 1 (capturing the variation in zinc, copper and manganese) implies that the underlying normalized soil nutrient variables each increase by one as well. Or, by virtue of the normalization (see section 3.2), each of the soil nutrient variables increases by one standard deviation. We make some simplifying assumptions. First, we ignore the other, smaller factor loadings, thus only calculating the costs for a standard deviation increase in zinc, copper and manganese. Second, we value these elements by considering recent price data from the London Metal Exchange. We thereby ignore all possible transaction costs involved in getting these elements included in inorganic fertilizers and transported to and applied at farmers’ fields. Moreover, not all fertilizer-applied nutrients may directly be available for plant nutrition. In other words, the cost estimate is a lower bound of the actual costs involved. Table 9 shows the calculation of the costs involved (i.e. the lower bound as per the discussion above) of raising the soil nutrient densities in one grid cell (of 5km x 5km) by one standard deviation. Taken together these costs amount to a little less than USD 80,000 per grid cell using price data from the London Metal Exchange [47].

**Table 9:** Cost estimate of increasing soil nutrient contents in a grid cell by one standard deviation.

	Standard deviation soil nutrients	Standard deviation increase per grid cell (25 km^2^)	Unit price	Total value of standard deviation increase
	(kg ha^-1^)	(1,000 kg)	(USD 1,000 kg^-1^)	(USD)
Copper (Cu)	0.86	2.145	5,710	12,248
Manganese (Mn)	22.56	56.395	1,053	59,384
Zinc (Zn)	1.18	2.957	2,785	8,238
Total across three micronutrients				79,870

Subsequently, the regressions in the previous section provide insights into the magnitude of the benefits. Recall from Table 8 that unit increase of factor 1 (Cu-Mn-Zn) results in a decrease of child mortality by 4,105 (at the median of the malaria stability index). Average (between one and five year) child mortality is around 44, thus a unit increase in soil factor one leads to a decrease in mortality from 44 to around 40 children per 1,000 children surviving their first birthday. We consider the median of the population density to translate this figure into actual child lives saved. The median of the population density is 9.96 persons per km^2^ (249 persons per 25km^2^ grid cell) and across Sub-Saharan Africa on average 16% of the population is aged between 0 and 4 years [48], 2015 estimates) (40 per grid cell). The mortality rate (44 per mille) implies that 1.64 children per grid cell are likely to pass away between their first and fifth birthday. Thus, a unit increase in soil factor 1 may reduce this figure by around 0.16 child lives (4 per mille points).

In the final step, we convert this figure to Disability Adjusted Life Years (DALY) saved by using the standard WHO formula: DALY = Years of Life Lost (YLL) + Years Lived with Disability (YLD). As we focus on reductions in child mortality we calculate YLL only, by using: $YLL=\frac{N}{r}\left(1-e^{-rL}\right)$, with *N* the number of deaths, *r* the discount rate (0.03) and *L* the standard life expectancy at the age of death [49]. The latter is set at 57 as we assume an average age of death (within the 0–4 age category) of 3, while the average life expectancy in Africa is 60 [50].Under these assumptions 0.16 child life saved equals 4.47 DALYs. Finally, considering the investment of USD 79,870 in soil nutrients, the costs per DALY equal USD 17,866. These calculations are summarized in the first row of Table 10.

**Table 10:** Child lives saved due to increasing soil nutrient content (zinc, copper and manganese).

Percentile at the population distribution	Population density	Population per grid cell	Children (0–4) per grid cell	Children saved by one standard deviation increase in soil nutrient density	Disability Adjusted Life Years (DALY) saved	Costs per DALY saved
	# per km^2^	# per 25 km^2^	#	#	#	USD DALY^-1^
50%	10	249	40	0.16	4.47	17,866
75%	31	787	126	0.52	14.11	5,661
95%	156	3,910	626	2.57	70.12	1,139
98%	293	7,328	1,173	4.81	131.43	608

Table provides insights into the costs per child life saved by increasing soil micronutrient densities as calculated in Table 8, for different percentiles of the population distribution.

The subsequent rows in Table 10 display similar calculations carried out for different points at the distribution of the population density. The costs per DALY saved decrease substantially for higher population densities. For instance, at the 95% percentile of the population density the costs are estimated at USD 1,139 per DALY saved. Even though such grid cells are relatively densely populated, they are still distinctively rural. Various studies define urban areas as those region with a population density of over 400 inhabitants per km^2^. In this sample, this implies all areas from just below the 99% percentile of the population distribution and upward. Areas that fall in this 95% - 98% percentile range are thus often close to major urban areas, but still with considerable intensive agricultural activity. Marked by relatively low transport costs for agricultural in- and outputs and, by consequence, higher use of inorganic fertilizers (e.g.[51]), such areas may also be best suitable for projects aimed at promoting inorganic fertilizers enriched with micronutrients. Simultaneously, such a strategy has the benefit of raising agricultural productivity in regions where food demand is highest, the benefits of which are not included in the cost-benefit assessment above.

**Fig 6:**
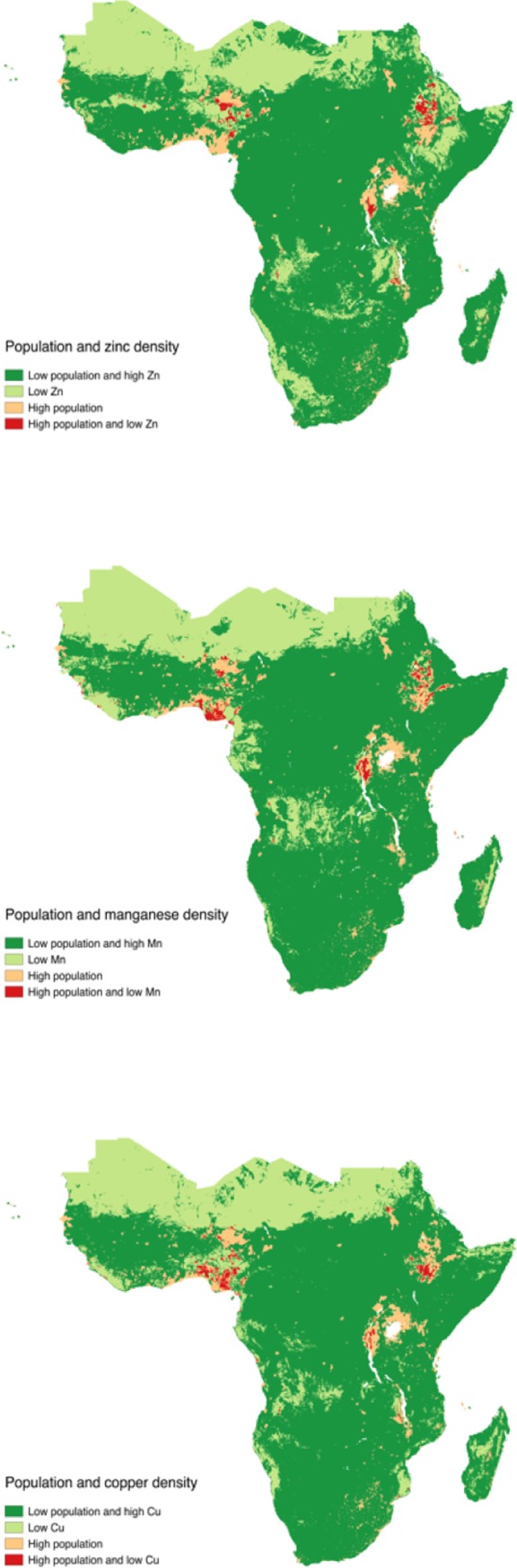
Regions with low micronutrient densities, high population densities, or both. The figure shows regions where soil densities of zinc (panel a), manganese (panel b) or copper (panel c) are low (<25% percentile) and population density is high (>95% percentile). Data on zinc, manganese and copper densities are from Hengl et al. [18], data on population density is from Center for International Earth Science Information Network—CIESIN—Columbia University [33].

**Fig 7:**
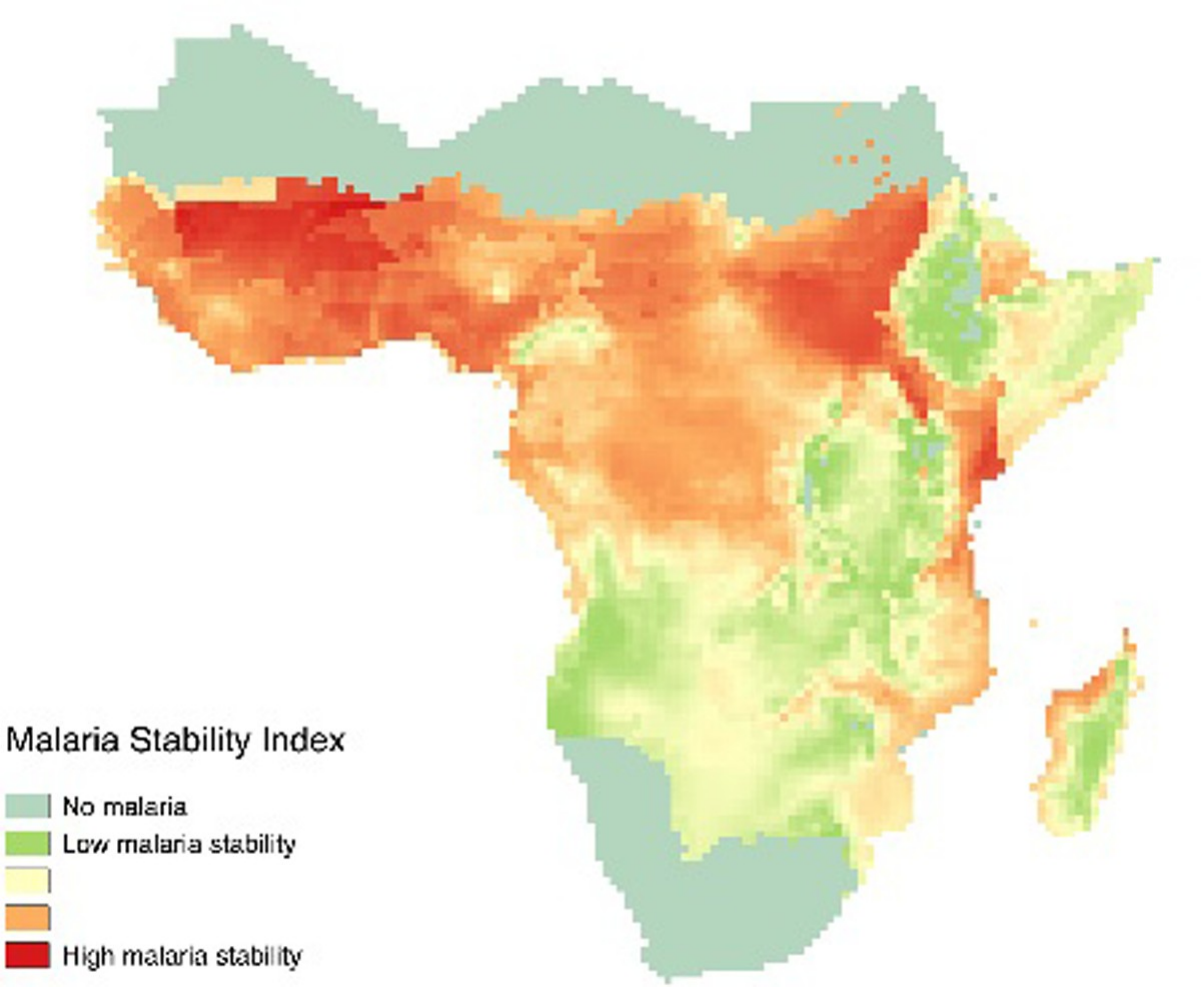
Variation in malaria pressure across Sub-Saharan Africa. Figure shows variation in malaria pressure based on the malaria stability index developed by Kiszewski et al. [35]. A low malaria stability index (in green) indicates low malaria pressure.

Thus, regions where the potential for agronomic fortification is greatest (high population densities and low soil nutrient densities) are shown in Fig 6A–6C. Across the three panels (showing the cases for zinc, manganese and copper in panel a, b and c respectively) the maps point to similar areas: in particular, the Ethiopian highlands, Rwanda and Burundi, and much of Nigeria and southern Niger. Furthermore, the results in Tables 6 and 7 highlight the negative impact of high malaria pressure and whereby the link between health and soils mostly exists in areas with low malaria pressure. In other words, agronomic fortification is likely to have an impact only in these areas with low malaria pressure. These, somewhat unsurprisingly, overlap with high population areas (Fig 7). Nevertheless, malaria pressure is considerably high in Southern Niger and Nigeria, rendering agronomic fortification potentially less effective.

Nevertheless, the range of the cost per DALY saved in more densely populated rural areas (156–293 inhabitants per km^2^)–roughly between USD 600 and 1,000– puts agronomic fortification at the higher cost end, compared with those of available alternatives. Table 10 shows an overview of costs per DALY per averted as compiled in the recent review by Gregory et al. [52], focusing on options to alleviate zinc and iron deficiencies. The first four rows list various fertilizer approaches, the first two based on the *ex ante* assessment by Joy et al. [12] on micronutrient enriched fertilizers for Sub-Saharan Africa. Their cost ranges (USD 81–575 and USD 773–6,457) overlap with those estimated above. Either way these ranges, and ours, remain high when compared with alternatives like crop breeding (genetic fortification). Indeed, for genetic fortification the costs per DALY averted are assumed to be well below USD 100 for many nutrient-crop combinations [9]. That said, as argued earlier, genetic fortification may have limited potential when inherent densities of nutrients in soil are low to begin with. The ranges for food supplementation and fortification, as shown in Table 11 (from Gregory et al. [52], may equally be on the high side. Another recent study, though not focusing exclusively on iron or zinc, puts the cost of saving a life of an infant or young child through micronutrient fortification and supplementation at USD 100 and 200 [53]. Using the assumptions underlying Table 10, this translates to roughly USD 1–3 per DALY averted.

**Table 11:** Estimated costs per DALY saved for a range of food system approaches to alleviate Zn and Fe deficiencies.

Intervention	Cost per DALY saved (USD)	Notes	Source
Granular fertilizer	773–6,457	Sub-Saharan Africa	Joy et al. [12]
Foliar fertilizer	81–575	Sub-Saharan Africa	Joy et al. [12]
Soil + foliar fertilizer	256–549	Pakistan (Punjab and Sindh province)	Joy et al. [54]
Foliar fertilizer (with pesticide)	41–594	China	Wang et al. [55]
Crop breeding	0.7–7.3	India	Stein et al. [5]
Supplements	65–2,758	Prophylactic, 1–4 years	Fink and Heitner [56]
Flour fortification	401	Zambia, vitamin A, Fe, Zn	Fiedler et al. [57]

Table from Gregory et al. [52]

It is worth noting two possible assumptions that may inflate our cost estimates in Table 10. First, the high costs per grid cell are strongly driven by the high costs of manganese (see Table 9). Of course, it could be that the results of soil nutrients on health are not driven by the combination of the three nutrients included in soil factor 1, but that a single nutrient is responsible for changes in these health outcomes. Unfortunately, the strong correlation between copper, zinc and manganese makes it difficult to isolate such singular causes. If we assume, momentarily, that all changes in health outcomes can be attributed to, say, zinc alone (around 10% of the total costs in Table 9), then the costs per DALY averted drop to USD 1,746 at the median population distribution, or to USD 59 at the 98% percentile. But, except for the more densely populated and more intensively farmed areas, such estimates render agronomic fortification still less cost-effective than available alternatives.

A second reason is that we may overstate the costs for grid cells with low population densities. In these areas, the actual area allocated to agricultural production could be smaller than the full grid cell size. That said, putting the cost range of USD 17,465 (at the median) within a reasonable range of alternatives would imply an assumption on the agricultural share in such grid cells to fall below 1%. This, we consider greatly unrealistic. Moreover, we should reiterate that other reasons may actually understate the costs estimates. Like stated we do not account for product development costs, neither for transport and other market transaction costs. In fact, such costs are bound to be higher in areas with relatively low population densities.

## Conclusion

This paper reveals a significant link between spatial variation in African soil fertility and spatial variation in child health. We are not aware of earlier studies that have identified such a relation in statistical detail. In establishing this link we use estimates [18] on densities of nine macro- and micronutrients, as well as estimates on soil organic matter content, to proxy for variation in soil fertility. We subsequently assess the precise causal effect of spatial variation in these elements on incidences of child mortality, stunting wasting and underweight. We argue that these effects serve as a good proxy for assessing the magnitude of the impact of agronomic fortification -enriching inorganic fertilizers with micronutrients- as a means to alleviate malnutrition or hidden hunger.

We find that joint increases in micronutrients (zinc, copper and manganese) lead to significant reductions in incidences of child mortality, stunting, wasting and underweight, specifically in areas where malaria pressure is relatively low. In regions with higher malaria pressure the effect dissipates. Greater (joint) densities of soil calcium and magnesium lead to taller children, all other things being equal, leading to increases incidences of child wasting and underweight, but to reductions in the incidences of child stunting. Finally, joint increases in soil nitrogen and organic matter content reduce incidence of child mortality and wasting. Overall the magnitude of these effects is noteworthy. A joint standard deviation increase in zinc, copper and manganese densities reduces child mortality by 4 per mille points at the median malaria intensity (from an average mortality rate of 44 per mille). For relatively low malaria pressure (1^st^ quartile) such a micronutrient increase leads to reductions in stunting, wasting and underweight of around 2–3 percentage points (from averages of 39, 10 and 21 percentage respectively). In other words, you are what you eat, at least in the case of Sub-Saharan Africa, which in turn is also shaped by local soil conditions.

These findings do suggest that building up soil nutrient stocks through agronomic fortification has its merit in reducing malnutrition and improving health. Various recent studies already document the benefits of including micronutrients in regular inorganic fertilizers as a means to increase agricultural production and productivity [10, 16] These effects on improving child health may thus be considered a co-benefit or external effect. These co-benefits inhibit a strong public good nature. Improved nutritional quality in agricultural produce is unlikely to attract a higher price in markets in SSA, next to various studies that reveal suboptimal individual spending on health goods and services to begin with. We are aware of only one study that links nutrient content of agricultural produce with actual farmer marketing decisions [58]. This study reveals that Ugandan farmers typically market produce with lower zinc content and self-consume produce with higher zinc content. In other words, these reasons may justify government subsidization of inorganic fertilizers enriched with missing (micro-)nutrients.

However, the cost-benefit analysis presented in the second half of this paper dampens the enthusiasm for presenting agronomic fortification as an omnipotent and efficacious instrument to combat malnutrition. We find that in many instances the costs per DALY averted are relatively high. Only in those areas where population density is high, as well as likely levels of agricultural intensification, is agronomic fortification likely to be a cost-effective alternative. This argument is shaped by both the fact that the per capita cost of agronomic fortification is lower in such areas, next to the fact that inorganic fertilizer use is more common to begin with. Conversely, in areas with lower population densities alternatives like nutrient supplementation (in capsules or powders), food fortification, possibly in combination with genetic fortification are likely to be more cost-effective.

Malnutrition continues to place a great burden on human health and development in Sub-Saharan Africa now and in the foreseeable future. The potential economic opportunities in reverting malnutrition are huge. But, solid information on cost and benefits of the various policy options, and how these differ, across regions and countries remain scarce. In filling this gap, this study made a contribution to better understand the potential for using agronomic fortification to revert malnutrition. Even though we argue that our approach provides realistic and robust insights into the potential of agronomic fortification, it remains second-best to urgently needed on the ground experimental approaches. Hence, the results from this study point to areas–high population density and low micronutrient densities- that most likely harbour the greatest potential for using agricultural fortification as a means to combat malnutrition. Such locations should be targeted first with actual field trials in which micronutrient enriched fertilizers are distributed and impact on both agricultural productivity and health indicators are monitored. Moreover, combining such trials with data collection on actual food consumption will allow for a better understanding on the precise causal channel through which changes in malnutrition may come about.

## Supporting information

S1 Dataset Data files and estimation procedures. (ZIP)

S1 Table Regression results with controls for night time lighting, population density and institutional hierarchy omitted. Robust standard errors in parentheses. *** p<0.01, ** p<0.05, * p<0.1.
(DOCX)

S2 Table Regression results for data aggregated at district level. Standard errors in parentheses. *** p<0.01, ** p<0.05, * p<0.1.
(DOCX)
