# Supplementary material for: Better soils for healthier lives? An econometric assessment of the link between soil nutrients and malnutrition in Sub-Saharan Africa
Source: PLoS One. 2019 Jan 17;14(1):e0210642. doi: 10.1371/journal.pone.0210642 (PMC6336299; doi:10.1371/journal.pone.0210642)
Supplement: S1 Table — Robust standard errors in parentheses. *** p<0.01, ** p<0.05, * p<0.1. (DOCX) [file pone.0210642.s002.docx]

**S1 Table. Regression results with controls for night time lighting, population density and institutional hierarchy omitted.**

|  | (1a) | (1b) | (2a) | (2b) | (3a) | (3b) | (4a) | (4b) |
| --- | --- | --- | --- | --- | --- | --- | --- | --- |
| VARIABLES | Child  Mortality | Child  Mortality | Child  Stunting | Child  Stunting | Child  Wasting | Child  Wasting | Child  Underweight | Child  Underweight |
| Cu–Mn–Zn | -3.676* | -6.390 | -1.433 | -2.671 | -2.283*** | -1.685 | -2.662** | -2.057 |
|  | (2.081) | (4.928) | (1.235) | (1.913) | (0.672) | (1.144) | (1.072) | (1.825) |
| Cu–Mn–Zn * Malaria Index |  | 0.210 |  | 0.106 |  | -0.0183 |  | 0.00379 |
|  |  | (0.283) |  | (0.104) |  | (0.0476) |  | (0.0862) |
| Ca–Mg | 2.726 | 3.367 | -2.010* | -3.679** | 2.496*** | 3.570*** | 3.421** | 4.711*** |
|  | (3.462) | (4.543) | (1.062) | (1.449) | (0.847) | (0.718) | (1.663) | (1.613) |
| Ca–Mg * Malaria Index |  | -0.108 |  | 0.139 |  | -0.0997* |  | -0.128 |
|  |  | (0.220) |  | (0.103) |  | (0.0588) |  | (0.122) |
| N–OMC | -2.819 | -1.292 | 1.150 | 3.818*** | -1.767*** | -1.540*** | -1.057 | 0.356 |
|  | (2.390) | (2.782) | (1.108) | (0.958) | (0.528) | (0.350) | (1.064) | (0.641) |
| N–OMC * Malaria Index |  | -0.146 |  | -0.307*** |  | -0.0196 |  | -0.147 |
|  |  | (0.245) |  | (0.104) |  | (0.0465) |  | (0.0876) |
| Malaria Index | 1.129*** | 1.106*** | -0.116 | -0.112 | 0.262*** | 0.229*** | 0.315*** | 0.254** |
|  | (0.349) | (0.343) | (0.113) | (0.108) | (0.0617) | (0.0578) | (0.0984) | (0.103) |
| Distance to capital (log) | 0.656 | 0.657 | 2.989*** | 3.321*** | 0.775* | 0.911** | 1.448** | 1.811*** |
|  | (1.477) | (1.192) | (0.887) | (0.744) | (0.452) | (0.434) | (0.629) | (0.638) |
| Distance to coast (log) | 5.226*** | 4.953*** | 0.475 | 0.195 | 0.798* | 0.737* | 1.753** | 1.498* |
|  | (0.981) | (1.001) | (0.977) | (0.828) | (0.455) | (0.419) | (0.820) | (0.757) |
| Distance to border (log) | -0.975 | -0.943 | 0.418 | 0.630** | 0.0794 | 0.0363 | 0.170 | 0.184 |
|  | (0.637) | (0.601) | (0.314) | (0.292) | (0.128) | (0.126) | (0.295) | (0.248) |
| Landlocked (dummy) | -2.557 | -2.939 | 4.065** | 3.101* | 1.317 | 1.282 | 2.675 | 2.260 |
|  | (5.645) | (5.738) | (1.779) | (1.802) | (1.572) | (1.542) | (2.745) | (2.544) |
|  |  |  |  |  |  |  |  |  |
| Observations | 622,017 | 622,017 | 514,409 | 514,409 | 494,405 | 494,405 | 494,405 | 494,405 |
| R-squared | 0.221 | 0.227 | 0.184 | 0.252 | 0.337 | 0.348 | 0.254 | 0.276 |

Robust standard errors in parentheses**.** *** p<0.01, ** p<0.05, * p<0.1
