# Supplementary material for: Better soils for healthier lives? An econometric assessment of the link between soil nutrients and malnutrition in Sub-Saharan Africa
Source: PLoS One. 2019 Jan 17;14(1):e0210642. doi: 10.1371/journal.pone.0210642 (PMC6336299; doi:10.1371/journal.pone.0210642)
Supplement: S2 Table — Standard errors in parentheses. *** p<0.01, ** p<0.05, * p<0.1. (DOCX) [file pone.0210642.s003.docx]

**S2 Table. Regression results for data aggregated at district level.**

|  | (1a) | (1b) | (2a) | (2b) | (3a) | (3b) | (4a) | (4b) |
| --- | --- | --- | --- | --- | --- | --- | --- | --- |
| VARIABLES | Child  Mortality | Child  Mortality | Child  Stunting | Child  Stunting | Child  Wasting | Child  Wasting | Child  Underweight | Child  Underweight |
| Cu–Mn–Zn | -8.074** | -3.693 | -3.556 | -13.09*** | -1.630 | -2.522 | -2.081 | -5.573 |
|  | (3.404) | (6.180) | (2.132) | (4.484) | (1.095) | (1.900) | (1.826) | (3.381) |
| Cu–Mn–Zn * Malaria Index |  | -0.460 |  | 0.734** |  | 0.0811 |  | 0.280 |
|  |  | (0.427) |  | (0.324) |  | (0.0861) |  | (0.203) |
| Ca–Mg | 4.201 | 0.588 | -1.366 | -4.328 | 3.632*** | 5.368*** | 6.734*** | 8.532*** |
|  | (4.001) | (4.429) | (1.993) | (3.324) | (1.004) | (0.991) | (2.194) | (2.512) |
| Ca–Mg * Malaria Index |  | 0.424 |  | 0.249 |  | -0.169** |  | -0.195 |
|  |  | (0.388) |  | (0.256) |  | (0.0821) |  | (0.192) |
| N–OMC | -2.567 | -2.838 | -0.0880 | 1.046 | -1.858*** | -2.943*** | -2.311** | -3.649*** |
|  | (1.950) | (3.109) | (1.088) | (1.703) | (0.517) | (0.750) | (1.067) | (1.208) |
| N–OMC * Malaria Index |  | 0.0272 |  | -0.161 |  | 0.105** |  | 0.119 |
|  |  | (0.277) |  | (0.170) |  | (0.0491) |  | (0.0859) |
| Institutional hierarchy | -2.238 | -1.840 | 1.559 | 0.855 | 0.625 | 0.512 | 0.212 | -0.0893 |
|  | (1.438) | (1.619) | (1.202) | (1.088) | (0.597) | (0.548) | (0.867) | (0.768) |
| Malaria Index | 0.986*** | 1.165*** | -0.206 | -0.00908 | 0.339*** | 0.259*** | 0.383*** | 0.300** |
|  | (0.241) | (0.356) | (0.174) | (0.139) | (0.0542) | (0.0666) | (0.0868) | (0.143) |
| Population density (logs) | 1.632 | 1.275 | 1.013 | 0.843 | -0.226 | 0.0980 | 0.218 | 0.666* |
|  | (1.052) | (0.965) | (0.749) | (0.553) | (0.241) | (0.278) | (0.409) | (0.388) |
| Night time luminosity (level) | -0.303* | -0.277* | -0.278* | -0.262** | 0.0484 | 0.0116 | -0.0707 | -0.125 |
|  | (0.163) | (0.163) | (0.161) | (0.117) | (0.0414) | (0.0421) | (0.0841) | (0.0781) |
| Distance to capital (log) | 3.101*** | 2.691** | 4.056*** | 3.783*** | 0.540 | 0.633 | 2.205** | 2.257*** |
|  | (1.051) | (1.074) | (0.934) | (0.835) | (0.449) | (0.398) | (0.833) | (0.798) |
| Distance to coast (log) | 5.573*** | 6.057*** | 0.407 | -0.234 | 0.362 | 0.694** | 1.289 | 1.573* |
|  | (1.500) | (1.699) | (1.404) | (1.410) | (0.290) | (0.339) | (0.907) | (0.878) |
| Distance to border (log) | -3.190* | -3.883** | -0.00429 | 0.817 | -0.735 | -0.862* | -1.501 | -1.410 |
|  | (1.758) | (1.863) | (1.659) | (1.496) | (0.445) | (0.472) | (1.041) | (1.004) |
| Landlocked (dummy) | 3.834 | 3.563 | 2.480 | 2.043 | 0.0683 | 0.639 | 1.520 | 2.224 |
|  | (4.809) | (5.044) | (2.289) | (2.138) | (1.190) | (1.161) | (2.054) | (1.996) |
| Constant | -46.76* | -39.28 | -23.78 | -22.10 | 2.250 | -2.560 | -12.60 | -19.21 |
|  | (26.40) | (27.13) | (16.99) | (16.67) | (8.784) | (9.088) | (18.54) | (18.58) |
|  |  |  |  |  |  |  |  |  |
| Observations | 301 | 301 | 288 | 288 | 267 | 267 | 267 | 267 |
| R-squared | 0.314 | 0.325 | 0.305 | 0.361 | 0.435 | 0.455 | 0.392 | 0.412 |

Standard errors in parentheses**.** *** p<0.01, ** p<0.05, * p<0.1
